# Detection of Polycyclic Aromatic Hydrocarbons in Water Samples by Annular Platform-Supported Ionic Liquid-Based Headspace Liquid-Phase Microextraction

**DOI:** 10.1155/2018/3765682

**Published:** 2018-09-27

**Authors:** Xiaojie Sun, Jie Tan, Haiyan Ding, Xiaojie Tan, Jun Xing, Lihong Xing, Yuxiu Zhai, Zhaoxin Li

**Affiliations:** ^1^Key Laboratory of Testing and Evaluation for Aquatic Product Safety and Quality, Ministry of Agriculture and Rural Affairs, Yellow Sea Fisheries Research Institute, Chinese Academy of Fishery Sciences, Qingdao 266071, China; ^2^Qingdao National Laboratory for Marine Science and Technology, Qingdao 266000, China; ^3^The Affiliated Hospital of Qingdao University, Qingdao 266000, China; ^4^Key Laboratory of Analytical Chemistry for Biology and Medicine, Ministry of Education, College of Chemistry and Molecular Sciences, Wuhan University, Wuhan 430072, China

## Abstract

In this paper, a new method of annular platform-supported headspace liquid-phase microextraction (LPME) was designed using ionic liquid as an extraction solvent, wherein extraction stability and efficiency were improved by adding an annular platform inside the extraction bottle. The ionic liquid 1-silicyl-3-benzylimidazolehexafluorophosphate was first synthesized and proved to be an excellent extraction solvent. Coupled with liquid chromatography, the proposed method was employed to analysis of polycyclic aromatic hydrocarbons (PAHs) in water and optimized in aspects of extraction temperature, extraction solvent volume, extraction time, pH, stirring rate, and salt effect of solution. The results indicated that this method showed good linearity (*R*^2^ > 0.995) within 0.5 *µ*g·L^−1^ to 1000 *µ*g·L^−1^ for PAHs. The method was more suitable for extraction of volatile PAHs, with recoveries from 65.0% to 102% and quantification limits from 0.01 to 0.05 *µ*g·L^−1^. It has been successfully applied for detection of PAHs in seawater samples.

## 1. Introduction

PAHs have been included in the environmental control legislation by many agencies, such as the US Environmental Protection Agency (EPA) [1] and the European Union. The maximum admissible concentration of naphthalene (1200 ng·L^−1^), anthracene (100 ng·L^−1^), fluoranthene (100 ng·L^−1^), and benzo[a]pyrene (100 ng·L^−1^) for superficial water was restricted by the European Union [2, 3]. To better evaluate the impact of PAHs on ecosystems, an efficient and sensitive analysis method must be developed. However, PAHs are found at very low levels (ng·L^−1^ or pg·L^−1^) in environment samples. It is necessary to perform an enrichment step in sample preparation due to the lack of sensitivity obtained with conventional analytical instrumentation.

Sample pretreatment has been the bottleneck of analytical chemistry. In recent decades, LPME has been developed rapidly as a novel pretreatment technology [4–7], reducing analysis time and large amounts of toxic solvent. Due to the low miscibility with water [8–10], ionic liquids (ILs) have become a new extraction solvent for LPME [11,12] and been extensively applied in sample pretreatment prior to chromatography [13–24], overcoming the toxicity and reducing the consumption of ordinary organic solvents. In addition, characteristics of ILs can be tuned by a combination of different anions and cations for task-specific extraction of analytes in various solvent mediums.

According to extraction patterns, application of ILs in the LPME technology can be classified into the following: dispersive liquid-liquid microextraction (DLLME) [25], hollow-fiber LPME [26], and suspended single-drop microextraction (SDME). Dispersive LPME requires large extraction amount with limited extraction efficiency; hollow-fiber LPME features low recovery with poor reproducibility; SDME has drawn much concern owing to its high enrichment efficiency. However, research studies showed that the sample solution should be stirred continuously during SDME, so extraction droplets easily fall off, affecting extraction stability and reproducibility. Furthermore, the droplets' volume of SDME was restricted, although adding a silicon rubber tube [12], a polytetrafluoroethylene sleeve [15], or a plastic tube [27] on a syringe needle seriously limits the improvement of extraction efficiency. Therefore, the SDME device and pattern should be optimized to promote the development of SDME technology. Furthermore, ILs have played crucial roles in the aspect. 1-Octyl-3-methylimidazolehexafluorophosphate ([C_8_MIM]PF_6_) was first used as an SDME solvent coupled with liquid chromatography to extract PAHs and aniline compounds in water samples [12]. Since then, some other types of ILs were used as an extraction solvent for SDME [28].

In this study, an IL-based headspace LPME device was designed using the homemade IL 1-silicyl-3-benzylimidazolehexafluorophosphate ([SBnIM]PF_6_) as an extraction solvent. The device was optimized by adding an annular platform inside the extraction bottle; then, IL was slowly pressed dropwise down to the annular platform by a syringe. The developed method was named “annular platform-supported IL-based headspace LPME” (APS-IL-LPME). The method altered the suspension state of extracted droplets, overcame the influence of droplet gravity, and significantly improved extraction stability and efficiency. Meanwhile, the large extraction platform can flexibly regulate addition of extraction solvent to control extraction capacity and enrichment efficiency. Considering that fluoranthene and naphthalene showed larger partition coefficients in the system of [C_*n*_MIM][PF_6_]/water [9,29], liquid chromatography was combined with APS-IL-LPME to determine PAHs in water samples. Simultaneously, the IL [SBnIM]PF_6_ containing the benzyl group in the structure of cation was used as an extraction solvent, which will be more suitable for extraction of analytes including benzene rings. The influence of different parameters on extraction was investigated and optimized in aspects of extraction temperature, extraction solvent volume, extraction time, pH, stirring rate, and salt effect of solution. Moreover, the proposed method was compared with other preconcentration methods for determination of PAHs in water samples.

## 2. Materials and Methods

### 2.1. Apparatus and Reagents

A 2695 high-performance liquid chromatograph was equipped with an automatic sampler and a 2475 fluorescence detector (Waters Corp., US). An XW-80A vortex mixer (Shanghai Medical University Instrument Factory), a Milli-Q ultrapure water meter (Millipore Corp., US), and a Sorvall Biofuge Primo centrifuge (Thermo Fisher Scientific Corp., US) were used.

1-Benzylimidazole (99%) was purchased from Fluka Corp. (US). *γ*-Chloropropyl-trimethylsilane, naphthalene (99%, Nap), acenaphthene (98%, Acp), fluorene (98%, Flu), anthracene (98.5%, Ant), fluoranthene (98%, FL), and pyrene (98%, Pyr) were purchased from AccuStandard Corp. (US). Acetonitrile was purchased from Shanghai ANPEL Corp. (China). Other solvents and reagents were of analytical grade (SCRC, China). The water used in this experiment was redistilled. With a brown glass bottle, water samples were collected from seawater in Shazikou, Qingdao, Shandong Province, and preserved under 4°C. Concentrations of six kinds of standard PAH stock solutions were separately 1.0 mg·mL^−1^, which were dissolved in acetonitrile, and the mixed standard stock solution was diluted to 10.0 *µ*g·mL^−1^ by acetonitrile. Subsequently, the solutions were stored at −20°C and diluted into standard working solutions of different concentrations by acetonitrile as needed.

### 2.2. The Device of APS-IL-LPME

**Figure 1:**
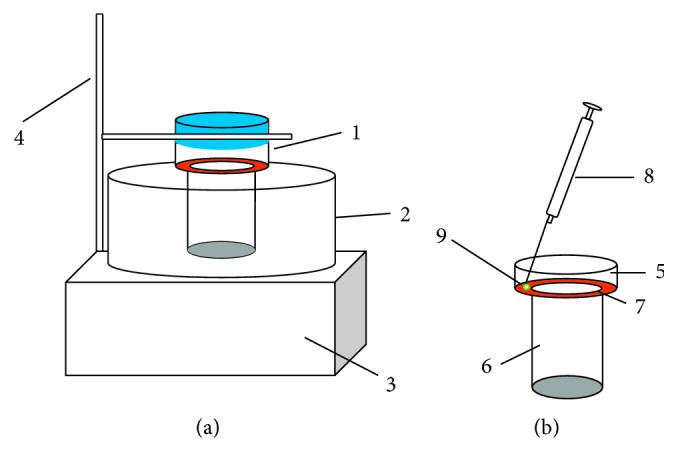
The device for APS-IL-LPME. (a) (1) Extraction bottle; (2) thermostatic water bath; (3) heating magnetic stirrer; (4) iron stand. (b) (5) The upper bottle; (6) the lower bottle; (7) annular extraction platform; (8) microsyringe; (9) ionic liquid droplets.

The APS-IL-LPME device was self-manufactured in the lab. As shown in Figure 1, the whole device includes a thermostatic water bath ((2) in Figure 1), a heatable magnetic stirrer ((3) in Figure 1), and an extraction bottle with a sealing bottle cap ((1) in Figure 1). The thermostatic water bath is laid on the heatable magnetic stirrer, and the extraction bottle (made of glass) with a sealing bottle cap (made of polytetrafluoroethylene) is placed in the thermostatic water bath and fixed on an iron stand. The extraction bottle mainly consisted of the upper bottle body (ID 4.0 cm, height 4.5 cm; (5) in Figure 1), annular extraction platform (width 0.75 cm; (7) in Figure 1), and lower bottle body (ID 2.5 cm, height 4.0 cm; (6) in Figure 1), all of which are integrated as a whole from top to bottom. Inner diameter of the upper bottle body is larger than that of the lower bottle body, and the annular extraction platform is a platform with high inner and outer sides and a sunken middle part, which is used to bear the weight of extracted droplets.

### 2.3. Preparation of ILs

**Figure 2:**
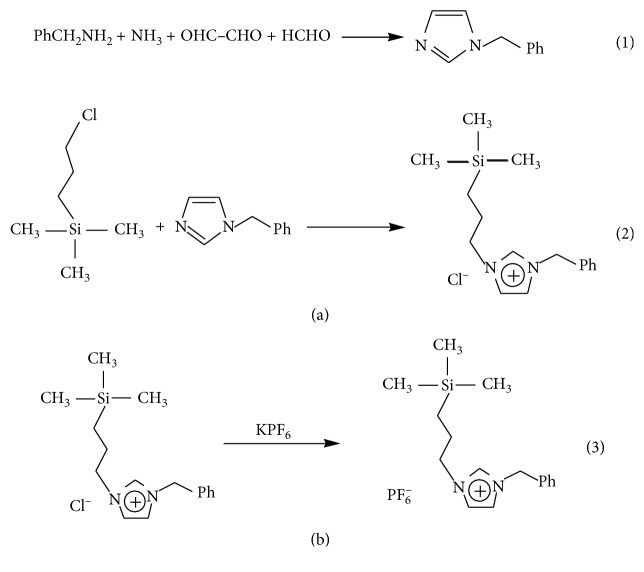
Synthetic routes of the ionic liquid [SBnIM]PF_6_.

The IL [SBnIM]PF_6_ was prepared as shown in Figure 2. Firstly, 1-benzylimidazolium was synthesized [30]. Then, [SBnIM]Cl (A) was prepared through a reaction between *γ*-chloropropyl-trimethylsilane and benzylimidazolium, which was according to the references [31,32]. Lastly, the chloride anion was exchanged for the hexafluorophosphate anion (PF_6_^−^) [33].

The general procedures for the synthesis of 1-benzylimidazole were as follows: formaldehyde water solution (36%, 8.35 g) and glyoxal water solution (32%, 18.1 g) were added to a 250 mL, three-necked flask provided with a stirrer and a reflux condenser. While the mixture was heated to 50°C, a mixture of benzylamine solution (10.7 g in 50 mL methanol) and ammonia solution (28%, 6.05 g) was added dropwise. After the mixture refluxed for an additional 4 h at 50°C, the methanol and water were removed under reduced pressure. The residue was then concentrated and dried in a vacuum desiccator at 60°C. The product of 1-benzylimidazole was obtained with the yield about 75%.

Secondly, 1-benzylimidazole (0.05 mol, 4.60 g) was added dropwise to a solution of *γ*-chloropropyl-trimethylsilane (0.05 mol, 17.3 g in 25 mL toluene). The solution was then refluxed for 72 h under N_2_ atmosphere. The resultant was washed with deionized water to remove the residue of reactants. After concentrating and drying, the light-yellow transparent and viscous compound [SBnIM]Cl (A) was obtained and the yield was about 90%.

Then, the IL [SBnIM]PF_6_ (B) was prepared through anion-exchange reactions. The process was as follows: the compound [SBnIM]Cl (A) was dissolved in water, and then 1 M equivalent of KPF_6_ in water was added dropwise. After stirring for 24 h at room temperature, the resultant was dissolved in dichloromethane and washed with deionized water. The silver nitrate test was used to confirm absence of chloride. Finally, the product was concentrated and dried, forming a light-yellow viscous compound [SBnIM]PF_6_ (B), and the yield reached 90%.

The final product [SBnIM]PF_6_ was confirmed by proton nuclear magnetic resonance (^1^HNMR) spectroscopy and Fourier transform infrared (FT-IR) spectroscopy. (i) ^1^HNMR (D_2_O; 400 MHz; *δ* (ppm)): 8.76 (s, 1H), 7.42–7.43 (d, 2H), 7.33–7.41 (m, 5H), 5.28 (s, 2H), 4.09 (t, 2H), 3.44 (m, 9H), 1.83 (m, 2H), and 0.56 (m, 2H). (ii) FT-IR (KBr, *υ* (cm^−1^)): 3127 and 3059 [*υ*(Ar–H)]; 2958, 2932, and 2868 [*υ*(C–H)]; 1558, 1494, and 1452 [*υ*(C=C), *υ*(C=N)]; 1156 [*υ*(Si–C)]; 1083 and 1023 [*υ*(Si–O)]; and 836 [*υ*(P–F)].

### 2.4. Extraction Procedures

First, 10 mL of the PAH-spiked water sample (5.0 *µ*g·L^−1^) was transferred to the extraction bottle, and sodium chloride was added to saturated concentration. The solution was inside the lower bottle body and remained below the annular extraction platform, which was headspace LPME. The sealing bottle cap was opened; the front end of the microsyringe (100 *µ*L) was placed in the middle position of the annular extraction platform (as shown in Figure 1(b)). Then, the syringe was slowly pressed down to release ILs (50 *µ*L) on the annular platform. The sealing bottle cap was fastened down; extraction was then implemented through stirring with the required water bath temperature. After extraction, the sealing bottle cap was loosened, the microsyringe was directly used to absorb IL on the annular platform, the solution was diluted to 100 *µ*L using acetonitrile, and 10 *µ*L of the solution was absorbed through the automatic sampler for high-performance liquid chromatography (HPLC) analysis.

### 2.5. Analysis Conditions

The analytes were separated on a Waters PAHC_18_ chromatographic column (250 mm × 4.6 mm, ID 5 *µ*m, Waters Corp.) at a column temperature of 40°C. The sample injection volume was 10 *µ*L. The mobile phase consisted of acetonitrile (A) and water (B). The total run time was 20.0 min with a flow rate of 1.0 mL min^−1^. The gradient elution steps were as follows: 0–10.0 min, A increased from 60% to 100%, then remained at 100% until 16.0 min, and thereafter returned to 60% in 1.0 min, followed by re-equilibration time for 3.0 min. The parameters of emission wavelength (*λ*_em_) and excitation wavelength (*λ*_ex_) for PAHs on a fluorescence detector are shown in Table 1.

## 3. Results and Discussion

### 3.1. Optimization of Extraction Conditions

Extraction efficiency of ILs for the analytes was mainly influenced by several parameters. In this study, optimized parameters included volume of the extraction solvent, extraction temperature, extraction time, pH, stirring rate, and salt effect of solution. During optimization of parameters, the PAH-spiked water sample (5.0 *µ*g·L^−1^) was used as an extraction substrate. Extraction efficiencies of methods were evaluated through enrichment factors (EFs) and recoveries. Computational formulas were expressed as follows:

(1) $$\begin{array}{c} \text{EFs}=\frac{C_{t}}{C_{0}}=\frac{\left(n_{t}/v_{t}\right)}{C_{0}}, \end{array}$$

(2) $$\begin{array}{c} \text{recovery}=\left(\text{EFs}\times \frac{V_{t}}{V_{0}}\right)\times 100\%, \end{array}$$

where *C*_*t*_ and *n*_*t*_ refer to the concentration and molar quantity of PAHs in the extraction phase, respectively; *C*_0_ is the initial concentration of PAHs in the water sample before extraction; *V*_*t*_ corresponds to the final volume of the extraction phase; and *V*_0_ stands for the volume of the water sample before extraction.

### 3.2. Volume of ILs

**Figure 3:**
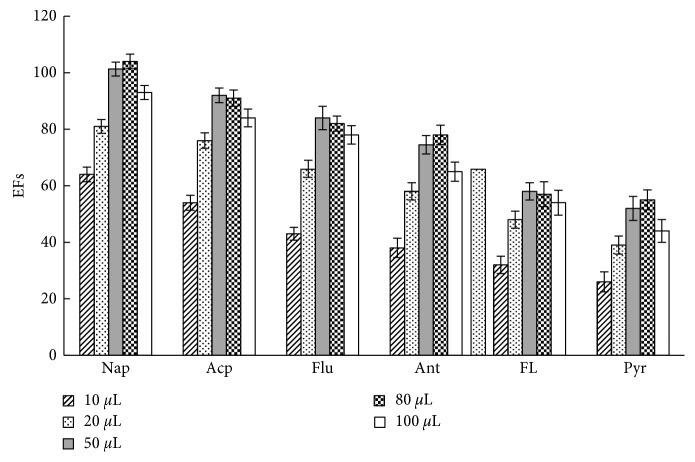
The effect of IL volume on EFs. Other extraction conditions: 10 mL of aqueous standards (containing PAHs at a concentration of 5 *μ*g·L^−1^); extraction temperature, 50°C; extraction time, 30 min; the concentration of NaCl, 25%. Values are mean of *n* = 3 replicates.

Volume of the extraction solvent is an important factor influencing extraction efficiency. This study assessed influences of different volumes (10, 20, 50, 80, and 100 *µ*L) of the IL [SBnIM]PF_6_ on extraction efficiency of six kinds of PAHs (Figure 3). When volume of IL was within 10 *µ*L to 50 *µ*L, extraction efficiency gradually improved as volume enlarged; enrichment efficiency remained within 50 *µ*L to 80 *µ*L but decreased when IL volume increased to 100 *µ*L. Thus, as volume of the extraction solvent enlarged, extraction amount (*n*_*t*_ in Formula (1)) of analytes correspondingly increased. However, when volume (*V*_*t*_) of the extraction solvent escalated too rapidly, analyte concentration (*C*_*t*_) and EFs decreased. Therefore, to obtain maximum enrichment efficiency and good reproducibility, volume of the IL extraction solvent was set at 50 *µ*L.

### 3.3. Extraction Temperature

**Figure 4:**
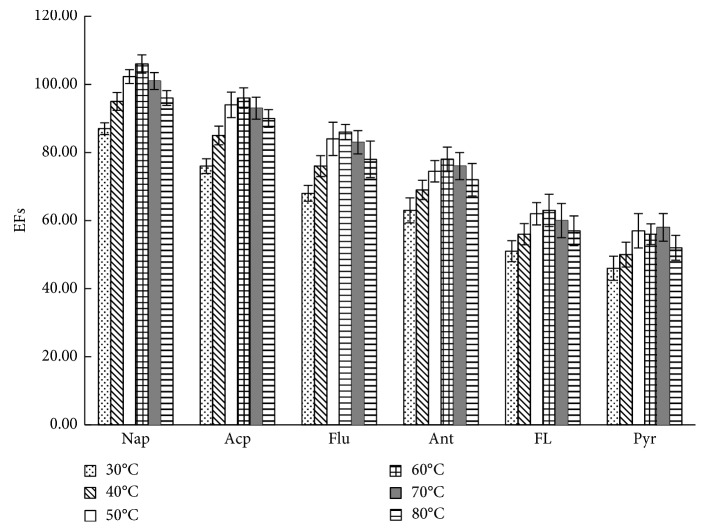
The effect of temperature on EFs. Other extraction conditions: 10 mL of aqueous standards (containing PAHs at a concentration of 5 *μ*g·L^−1^); extraction temperature, 50°C; extraction solvent volume, 50 *μ*L; extraction time, 30 min; the concentration of NaCl, 25%. Values are mean of *n* = 3 replicates.

To obtain satisfactory enrichment efficiency, the added water sample should not be higher than the level of the annular extraction platform. The extraction process involved headspace LPME. Extraction temperature directly influenced velocity and concentration of analytes released from the water sample and then influenced extraction efficiency indirectly. The increase in extraction temperature will shorten equilibrium time, accelerate analysis, and increase concentration of analytes in the gas phase. However, extraction for analytes was an absorption process. Thus, high temperature will reduce the distribution coefficient of analytes in the extraction solvent and also diminish extraction amount. Therefore, this study also assessed influences of different temperatures on extraction efficiency (Figure 4). When the temperature increased from room temperature to 50°C, EFs of six kinds of PAHs increased rapidly but slowed down under 50°C to 60°C and decreased under 70°C. Thus, in this study, 50°C was selected as an optimal extraction temperature.

### 3.4. Extraction Time

**Figure 5:**
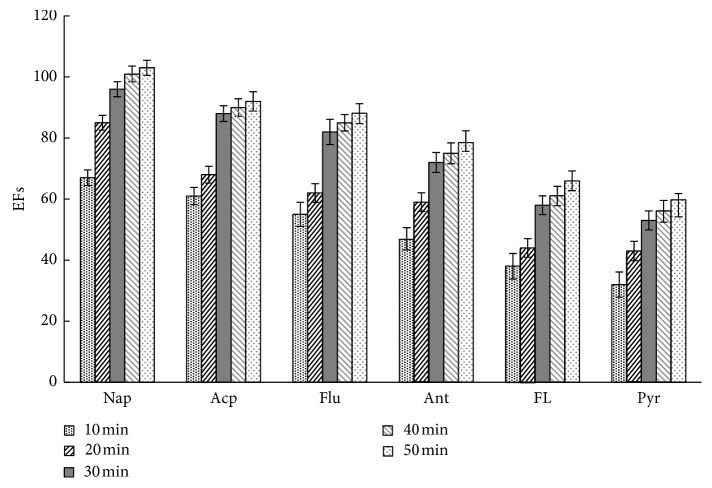
The effect of extraction time on EFs. Other extraction conditions: 10 mL of aqueous standards (containing PAHs at a concentration of 5 *μ*g·L^−1^); extraction temperature, 50°C; extraction solvent volume, 50 *μ*L; extraction time, 30 min; the concentration of NaCl, 25%. Values are mean of *n* = 3 replicates.

Theoretically, before reaching the equilibrium during extraction, prolonged duration will increase contents of analytes absorbed by the extraction solvent. This study also evaluated the influence of extraction time on extraction efficiency under 50°C (Figure 5). EFs of different kinds of PAHs improved rapidly before 30 min and improved slowly afterward. In this study, the extraction time was set to 30 min to guarantee EFs of analytes. However, according to Figure 5, if larger EFs are desired, an extraction time up to 50 min would be adopted.

### 3.5. pH of Solution

To study the influence of pH on extraction efficiency, 1 mol·L^−1^ HCl or NaOH was used to regulate pH of the extracted water sample within 2 to 13. The results indicated that pH exerted minor influence on extraction efficiency of PAHs. Maybe, it was due to the fact that PAHs had no ionizable groups and they were stable at the molecular state, a condition not influenced by pH of the solution. Therefore, for convenience and reproducibility, water samples can be directly extracted and enriched without any assistance only if their pH were within 2 to 13.

### 3.6. Stirring Rate

In order to evaluate the influence of stirring rate, 10 mL of 5 *μ*g·L^−1^ PAH mixed aqueous solution was extracted at 50°C for 30 min using 50 *μ*L [SBnIM]PF_6_ with stirring rates from 200 to 1500 rpm. Theoretically, before reaching the equilibrium, accelerating the stirring rate will increase contents of analytes absorbed by the extraction solvent. The results showed that extraction efficiency of all analytes slightly increased as the increase of stirring rate was up to 1000 rpm, while the reproducibility was poor after the stirring rate greater than 1000 rpm. A stirring rate of 1000 rpm was selected in this work.

### 3.7. Salt Effect

**Figure 6:**
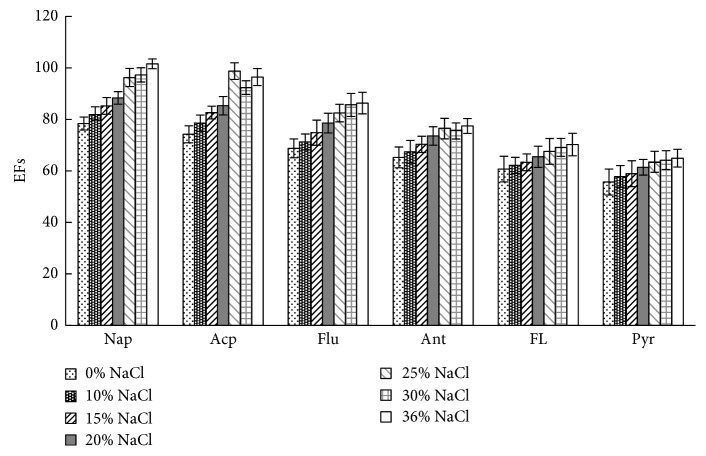
The effect of NaCl% on EFs. Other extraction conditions: 10 mL of aqueous standards (containing PAHs at a concentration of 5 *μ*g·L^−1^); extraction temperature, 50°C; extraction solvent volume, 50 *μ*L; extraction time, 30 min. Values are mean of *n* = 3 replicates.

In headspace LPME, adding NaCl in the sample solution can reduce solubility of analytes in the water phase, improve the gas-liquid partition coefficient, and enlarge concentration of analytes in the gas phase to increase extraction amount indirectly. However, extremely high NaCl concentration will hinder transport of analytes in the sample solution and influence their diffusion in the gas phase. This study assessed the effects of NaCl addition (0%–36%, w/v) on extraction efficiency (Figure 6). The results showed that enrichment efficiency was the highest when the NaCl solution was saturated, indicating that reducing solubility of PAHs in the water phase exerted a significant influence. Therefore, the optimum concentration for salt addition was saturated NaCl solution.

### 3.8. Method Evaluation

To evaluate practical applicability of the APS-IL-LPME method, reproducibility, limit of detection (LOD), and limit of quantification (LOQ) were investigated with PAH-spiked ultrapure water under the optimum conditions, the results of which are summarized in Table 2. It showed that the method presented good linearity (*R*^2^ > 0.995) within 0.5 *µ*g·L^−1^ to 1000 *µ*g·L^−1^ for the six kinds of PAHs. Replicate extractions of spiked ultrapure water solution (5.0 *µ*g·L^−1^) were carried out on intraday and interday to show precision of the method. The relative standard deviation (RSD) of PAHs ranged from 4.7% to 11.6% in all cases, and LOQ was within 0.01 *µ*g·L^−1^ to 0.05 *µ*g·L^−1^. The recoveries of Nap, Acp, Flu, Ant, and FL were within 70.2% to 102%, whereas only that of Pyr was lower than 70%. Meanwhile, EFs ranged from 65.0 to 102, which were comparable with those reported by Liu et al. [12].

The comparison of different methods for determination of PAHs in water is shown in Table 3. LLE [34] used less extraction time and exhibited low LOQ, but it was relatively solvent-consuming and had lower extraction efficiency. The SPE [35] method gave higher extraction efficiency. However, it exhibited high LOQ and needed more sample volume, while the operation of SPME [36] was complex and time-consuming. The LPME combined with gas chromatography [25, 37, 38] showed higher LOQs. The IL-SDME method [12] showed high EFs, while had lower extraction efficiency (<50%), calculated according to Formula (2) in Section 3.1. The previous reported IL-LPME methods [12, 22, 39, 40] all used [C_8_MIM]PF_6_ as an extraction solvent, and the LODs of most of them were higher than those of the proposed method. In conclusion, the proposed APS-IL-LPME method is simple, stable, and an advantageous alternative, the extraction efficiency and LOQ of which are better than or comparable with those of other methods.

### 3.9. Application to Real Water Samples

**Figure 7:**
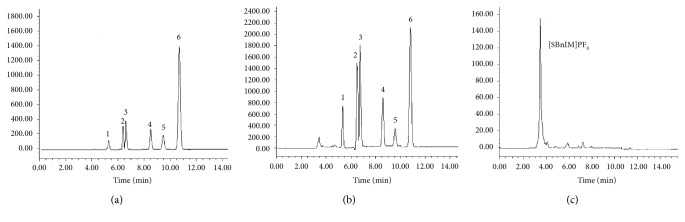
Chromatograms of (a) standard solution (200 *µ*g·L^−1^), (b) ionic liquid extracted spiked seawater (10 *µ*g·L^−1^), and (c) ionic liquid-extracted blank seawater. Peaks: (1) naphthalene; (2) acenaphthene; (3) fluorene; (4) anthracene; (5) fluoranthene; (6) pyrene.

Seawater samples collected from Shazikou in Qingdao and corresponding PAH-spiked seawater samples were extracted and detected according to the optimization method. As shown in Figure 7(c), no PAHs were found in the seawater sample. To evaluate this method, the seawater sample was spiked with PAHs at two levels (5.0 and 10 *µ*g·L^−1^). As can be seen in Table 4, the recoveries for six PAHs were from 67.2% to 106% with the RSD between 3.5% and 10.4%. Compared with the recoveries of PAHs in spiked ultrapure water solution, the effects of matrix constituents were minor.

Figure 7(b) shows the typical chromatogram of the sample spiking with 10 *μ*g·L^−1^ of the PAHs. After APS-IL-LPME, PAHs in seawater were enriched effectively, and the baseline was well separated. Combined with the results from Figures 3–6 and Table 4, EFs and recoveries decreased gradually with increasing boiling point and molecular weight due to decreased molecule diffusion and gas-liquid partition coefficient. The results were consistent with the findings of headspace LPME reported by other studies [12], indicating that this method is more suitable for enrichment of volatile PAHs in water samples due to the nonvolatile property of IL.

## 4. Conclusions

This work proposed a new method of APS-IL-LPME using the IL [SBnIM]PF_6_ as an extraction solvent, which overcame the influence of IL gravity and significantly improved extraction stability and efficiency. The IL [SBnIM]PF_6_ was homemade and proved to be an excellent extraction solvent for LPME. Combined with liquid chromatography, the method was employed to detect PAHs in the water sample. The optimized conditions for extraction were under 50°C for 30 min with 50 *µ*L extraction solvent and saturated NaCl concentration in the sample solution. The results indicated that the extraction efficiency and LOQ of the method were better than or comparable with the previously reported ones and suitable for detection of PAHs, such as Nap, Acp, Flu, Ant, FL, and Pyr, in water solutions, with EFs ranging from 65.0 to 102 and LOQ from 0.01 to 0.05 *µ*g·L^−1^. The APS-IL-LPME method was first used for enrichment of PAHs in real seawater samples, indicating that it will have broad potential in enrichment and analysis of organic pollutants in the environment and food samples. This method is especially advantageous for the enrichment of volatile compounds and shows higher extraction stability than suspended SDME by adding an annular platform. However, as the headspace LPME mode, the amount of water samples is limited and not higher than the level of annular platform. Moreover, in this work, nonvolatile compounds are not transferred to the headspace and not extracted by the ionic liquids. In the future work, we will continue to develop a new extraction mode to apply this method in immersed LPME for extraction of nonvolatile compounds.

## Figures and Tables

**Table 1:** The fluorescence detection wavelengths for PAHs.

Time (min)	*λ*_ex_ (nm)	*λ*_em_ (nm)
0	275	325
6.85	252	370
7.80	250	390
9.00	280	460
10.0	320	380
11.5	270	390

**Table 2:** Main analytical parameters for the enrichment of PAHs with the proposed method.

Analytes	Linear range (*μ*g·L^−1^)	*R* ^2^	LODs (*μ*g·L^−1^)	LOQs (*μ*g·L^−1^)	Precision (%RSD)	
					Intraday (*n* = 6)	Interday (*n* = 3)
Nap	0.5–1000	0.9981	0.003	0.01	4.7	7.9
Acp	0.5–1000	0.9976	0.003	0.01	4.8	7.4
Flu	0.5–1000	0.9954	0.006	0.02	5.1	8.1
Ant	0.5–1000	0.9988	0.010	0.03	5.5	10.7
FL	0.5–1000	0.9963	0.015	0.05	6.8	11.6
Pyr	0.5–1000	0.9972	0.003	0.01	5.3	9.6

**Table 3:** Comparison of the proposed method with other preconcentration methods for determination of PAHs in water.

Preconcentration technique	Analytical technique	Extraction solvent	Sample volume (mL)	Solvent volumea (mL)	Extraction time (min)	Extraction efficiency (%)	LOQ (*µ*g·L^−1^)	Reference
LLE	HPLC-Flu	CH_2_Cl_2_	250	90	6	51–104	0.1–4.4	[34]
SPE	HPLC-Flu	C_6_H_14_-CH_2_Cl_2_	50	5	50	67–99	0.7–56.2	[35]
SPME	GC-MS/MS	—	18	—	70	60–102	0.1–1.0	[36]
DLLME	GC-FID	C_2_Cl_4_	5	0.008	Seconds	60–111	23.3–100.0	[25]
LLME-FA	GC-FID	Toluene	22	0.05	5	100–108	14–41 (LOD)	[37]
IL-DLLME	GC-MS	[P_6,6,6,14_^+^][Ni(II)(hfacac)_3_^−^]	25	0.025	10	84–115	0.5–8.7 (LOD)	[38]
IL-DLLME	HPLC-UV	[C_8_MIM]PF_6_	10	0.055	30	83.5–118	0.0005–0.88 (LOD)	[39]
IL-DLLME	HPLC-Flu	[C_8_MIM]PF_6_	10	0.05	2	90–102	0.1–7.0	[22]
IL-SDME	HPLC-Flu	[C_8_MIM]PF_6_	15	0.003	30	—	—	[12]
IL-HS-LPME	HPLC-Flu	[C_8_MIM]PF_6_	5	0.003	60	88–110	0.03–0.1 (LOD)	[40]
APS-IL-LPME	HPLC-Flu	[SBnIM]PF_6_	10	0.05	30	65.0–102	0.01–0.05 (LOQ)/0.003–0.015 (LOD)	This work

—, not required or not available. ^a^The volume of extraction solvent needed in the extraction process.

**Table 4:** Spiked recoveries of the six PAHs in real seawater samples.

Analytes	Spiked amounts (*µ*g·L^−1^)	Average recovery (%)	RSD (%) (*n* = 5)
Nap	5	106	4.2
	10	105	3.5
Acp	5	97.8	5.5
	10	95.3	7.5
Flu	5	86.2	9.7
	10	81.4	6.1
Ant	5	77.3	6.3
	10	79.8	6.0
FL	5	72.5	6.5
	10	70.3	4.9
Pyr	5	68.3	10.4
	10	67.2	8.2

## Data Availability

The data used to support the findings of this study are included within the article.
